# An examination of auditory processing and affective prosody in relatives of patients with auditory hallucinations

**DOI:** 10.3389/fnhum.2013.00531

**Published:** 2013-09-06

**Authors:** Rachel Tucker, John Farhall, Neil Thomas, Christopher Groot, Susan L. Rossell

**Affiliations:** ^1^School of Psychological Sciences, La Trobe UniversityMelbourne, VIC, Australia; ^2^Brain and Psychological Sciences Research Centre, Swinburne UniversityMelbourne, VIC, Australia; ^3^Monash Alfred Psychiatry Research Centre, The AlfredMelbourne, VIC, Australia; ^4^School of Psychological Science, University of MelbourneMelbourne, VIC, Australia

**Keywords:** auditory hallucinations, schizophrenia, auditory perception, affect recognition, first-degree relatives

## Abstract

Research on auditory verbal hallucinations (AVHs) indicates that AVH schizophrenia patients show greater abnormalities on tasks requiring recognition of affective prosody (AP) than non-AVH patients. Detecting AP requires accurate perception of manipulations in pitch, amplitude and duration. Schizophrenia patients with AVHs also experience difficulty detecting these acoustic manipulations; with a number of theorists speculating that difficulties in pitch, amplitude and duration discrimination underlie AP abnormalities. This study examined whether both AP and these aspects of auditory processing are also impaired in first degree relatives of persons with AVHs. It also examined whether pitch, amplitude and duration discrimination were related to AP, and to hallucination proneness. Unaffected relatives of AVH schizophrenia patients (*N* = 19) and matched healthy controls (*N* = 33) were compared using tone discrimination tasks, an AP task, and clinical measures. Relatives were slower at identifying emotions on the AP task (*p* = 0.002), with secondary analysis showing this was especially so for happy (*p* = 0.014) and neutral (*p* = 0.001) sentences. There was a significant interaction effect for pitch between tone deviation level and group (*p* = 0.019), and relatives performed worse than controls on amplitude discrimination and duration discrimination. AP performance for happy and neutral sentences was significantly correlated with amplitude perception. Lastly, AVH proneness in the entire sample was significantly correlated with pitch discrimination (*r* = 0.44) and pitch perception was shown to predict AVH proneness in the sample (*p* = 0.005). These results suggest basic impairments in auditory processing are present in relatives of AVH patients; they potentially underlie processing speed in AP tasks, and predict AVH proneness. This indicates auditory processing deficits may be a core feature of AVHs in schizophrenia, and are worthy of further study as a potential endophenotype for AVHs.

## Introduction

Auditory verbal hallucinations (AVHs) are a phenomenon in which people experience hearing speech in the absence of appropriate sensory stimulation and with the full sense of reality of a true perception. Although occurring in a range of populations, AVHs are most frequently associated with schizophrenia, affecting approximately 75% of the schizophrenia population during a 1-year period (Bauer et al., 2011). This paper examines the role of auditory processing in AVHs, and examines whether difficulties with auditory processing are a core difficulty also observable in the relatives of persons with AVHs.

Auditory deficits have frequently been found in persons with schizophrenia (Cooper, 1976; Rabinowicz et al., 2000; Veuillet et al., 2001; Iliadou and Iakovides, 2003); with some studies establishing these deficits are more profound in patients with a history of AVH (Mckay et al., 2000). Neurophysiological literature has also established auditory deficits in schizophrenia. Mismatch negativity (MMN) is a negative polarity element of an event-related potential (ERP) that typically occurs from 100 to 200 ms after stimulus onset, when a deviant sound is perceived among a homogenous set (Näätänen et al., 1978). MMN is said to represent pre-attentive acoustic processing (Shinozaki et al., 2002). Impaired MMN (attenuated amplitude) in response to duration-deviant stimuli has been found in both schizophrenia patients (Baldeweg et al., 2002; Umbricht and Krljes, 2005) and unaffected relatives (Michie et al., 2002; Sevik et al., 2011). Further, attenuated MMN amplitude in response to pitch-deviant stimuli have also been observed in patients (Umbricht and Krljes, 2005) and relatives (Jessen et al., 2001). A number of recent studies have suggested that presence of AVH contribute to the pattern of MMN deficits (attenuated duration MMN amplitudes) in schizophrenia (Fisher et al., 2011, 2012).

It has been suggested that behavioral tasks that require participants to discriminate between two tones that differ in pitch are behavioral representations of neurophysiological deficits of pitch-deviant MMN (Javitt et al., 2000). Such pitch perception deficits have been found in schizophrenia patients (Leitman et al., 2005); a finding that has been consistently replicated (Leitman et al., 2006, 2010a; Matsumoto et al., 2006; Kantrowitz et al., 2013). This finding appears robust, regardless of the stage of illness, with deficits found in chronic inpatients, outpatients, and first episode schizophrenia patients (Rabinowicz et al., 2000). Further, pitch has been shown to be a key element that is manipulated to convey various emotions, along with amplitude and duration (Leitman et al., 2010b).

A number of studies have demonstrated that schizophrenia patients have difficulty perceiving and discriminating emotions based on affective prosody (AP) cues, compared with controls (Murphy and Cutting, 1990; Kerr and Neale, 1993; Rabinowicz et al., 2000; Edwards et al., 2001; Rossell and Boundy, 2005; Bozikas et al., 2006; Leitman et al., 2007, 2010a; Shea et al., 2007; Kantrowitz et al., 2013). Further, there is increasing evidence to suggest that AVH status is associated with schizophrenia patients' performance in AP detection and discrimination: AVH patients have been found to perform worse on AP tasks than non-AVH schizophrenia patients (Rossell and Boundy, 2005; Shea et al., 2007; Rossell et al., in press).

Some authors have theorized that AVHs could be the result of these developmental deficits in auditory sensory processing (Woodruff et al., 1997; Rossell and Boundy, 2005). It is further argued that these bottom-up sensory processes affect higher order cognitive processes, such as AP, required to comprehend emotion content of speech (Leitman et al., 2005). For example, recognition of AP requires accurate detection of variability in basic elements of auditory perception such as pitch, duration, and amplitude and deficits in these basic abilities would affect the ability to decode emotions based on AP.

An endophenotype is a characteristic associated with a particular illness, that occurs in non-affected family members at a higher rate than in members of the general population, and is not directly observable (Gottesman and Gould, 2003). Studying endophenotypes for schizophrenia is useful, as predisposing factors for the illness can be examined whilst confounding effects associated with psychosis, such as interactions with treatment and medication, long term unemployment, and hospitalization among other factors, can be controlled. Potential endophenotypes in schizophrenia have been found in cognition, including verbal memory, attention, and executive function (Sitskoorn et al., 2004). The most robust findings appear to be in impaired attention, as measured by the continuous performance task (Erlenmeyer-Kimling and Cornblatt, 1978; Nuechterlein, 1983; Appels et al., 2003; Birkett et al., 2007). However, potential emotion recognition endophenotypes have also been identified. Relatives of schizophrenia patients display similar impairments to patients in facial emotion recognition (Leppanen et al., 2008; Erol et al.) and on social cognition tasks (Anselmetti et al., 2009; de Achaval et al., 2010). Although, to date, these impairments have not been related to AVH proneness in the relatives; or examined for differences between relatives with a family member with a positive history of AVH vs. a negative history.

Whilst endophenotypes have been studied in relation to schizophrenia, there is no research examining potential endophenotypes specific to AVHs. Given observations of broader emotion perception deficits in relatives of people with a schizophrenia diagnosis, and specific auditory AP and associated basic auditory perception difficulties in patients with AVH compared to those without AVH; AP and associated auditory perception abilities are promising potential endophenotypes specific to AVHs worth investigation.

This study compared first degree relatives of schizophrenia patients with AVHs and controls in pitch, amplitude, duration, and AP perception. Based upon the strong findings of pitch perception deficits in schizophrenia (Leitman et al., 2005, 2006, 2010b; Kantrowitz et al., 2013), and preliminary evidence of similar reduced pitch MMN in relatives similar to that found in patients (Jessen et al., 2001), we first predicted that relatives would be less accurate than controls in pitch discrimination. In addition, exploratory comparisons were conducted between controls and relatives for amplitude and duration discrimination prompted by the absence of published studies examining these processes in relatives.

Given AVH schizophrenia patients exhibit deficits in AP (Rossell and Boundy, 2005), relatives were expected to perform less accurately in emotion identification based on AP. Given that difficulties with AP may be less pronounced in relatives, we additionally predicted that relatives would exhibit significantly slower reaction times (RTs) than controls when required to identify emotions based on AP. RT has been used as an important variable when investigating social cognitive processes, including AP perception (Green et al., 2008). Further, previous research has linked schizophrenia patients and their relatives to slower reaction time (RT) on tasks that require sustained attention (Birkett et al., 2007). If perceiving AP is difficult for an individual, one could assume their RT would be slower than for individuals who find the task easy. Therefore, RT is likely to function as an objective indicator of difficulty in perceiving AP, and was measured in addition to accuracy. Given prior findings of impaired attention in relatives, we examined whether RT on the AP task was independent of performance on attention and vigilance tasks.

We further wanted to explore whether acoustic processing deficits are related to psychosis proneness in general, or whether they are specific to AVHs. We hypothesized that within the overall sample, acoustic processing deficits would predict AVH proneness specifically, using an AVH-specific sub-factor derived from the Launay-Slade Hallucination scale—LSHS (Laroi and Van der Linden, 2005), but would not predict overall psychosis proneness with the AVH proneness items removed, which was examined using a broader measure of schizotypy.

## Materials and methods

### Participants

Thirty-three non-clinical controls (14 males and 19 females) and 19 first-degree relatives (4 males and 15 females) of schizophrenia patients who experience AVHs were recruited for this study, with an age range of 18–65 years. Relatives comprised parents, siblings, and offspring of schizophrenia patients who had experienced AVHs during the course of their illness. That is, had frequent AVH (almost all the time) and were classified as persons with chronic schizophrenia (illness durations of 5 years plus). Participants were recruited using the Monash Alfred Psychiatry Research Center (MAPrc) participant registry, advertisements, and convenience sampling.

Participants from both groups were excluded if they reported significant hearing impairment such as tinnitus, or failed a basic auditory threshold testing frequencies of 500, 1000, 1500, 2000, 3000, 4000, and 6000 Hz, with 25 dB used as the threshold of normal hearing for inclusion in the study. Controls were excluded if they currently suffered from an Axis I disorder, or if they had a first-degree relative with a psychotic disorder. Relatives were excluded if they had a history of a psychotic disorder, met criteria for schizophrenia, were taking antipsychotic medication, or if their relative with schizophrenia had never experienced AVHs in the course of their illness. Participants were paid a gratuity of $30 for their time and travel expenses.

### Measures

#### Mini international neuropsychiatric interview

To screen for Axis I disorders, as classified by the DSM-IV TR, a Mini International Neuropsychiatric Interview (MINI) screen and if necessary, MINI interview 5.0.0 (Sheehan et al., 1998) were administered to all participants. The MINI has been shown to have good inter-rater and test-retest reliability and has good concordance with DSM-IV diagnoses (Sheehan et al., 1998).

#### Oxford-liverpool inventory of feelings and experiences (O-LIFE)

The O-LIFE (Mason et al., 1995) was utilized to assess group differences in psychosis proneness. The four main scales of the O-LIFE are: unusual experiences (UnEx: this scale is thought to represent hallucination-proneness), cognitive disorganization (CogDis), introvertive anhedonia (IntAn), and impulsive non-conformity (ImpNon). Each of these scales are said to represent an element of schizotypy (Mason et al., 1995). A sub-factor of the O-LIFE comprising IntAn, CogDis, and ImpNon was created to measure psychosis proneness in the absence of hallucinatory elements (UnEx). This new sub-factor achieved a Cronbach alpha of 0.92, indicating it is a reliable measure for psychosis proneness; and is the measure used in the analysis.

#### Launay-slade hallucination scale (LSHS)—modified version

The modified version of the LSHS (Bentall and Slade, 1985) was used to measure hallucination proneness in the sample. However, this version of the LSHS contains some items that are not relevant for AVHs. Several factor structures were considered, but their hallucinatory components contained items relating to visual as well as auditory hallucinations (Aleman et al., 2001; Waters et al., 2003; Fonseca-Pedrero et al., 2010). Laroi and Van der Linden (2005) identified an auditory hallucination factor in their principal components analysis, with an Eigenvalue of 1.61. The LSHS hallucination factor (LSHS-HF) includes three items (“I have been troubled by hearing voices in my head”; “In the past, I have had the experience of hearing a person's voice and then found that no one was there”; and “I often hear a voice speaking my thoughts aloud”) with loadings of 0.73, 0.68, and 0.63, respectively. The maximum score for the sub-factor is 12. This factor was found to be reliable, with a Cronbach alpha of 0.76, and was used in the analysis as it appeared to be the most reliable sub-factor that isolated AVH symptoms from other hallucinatory items.

#### Wechsler test of adult reading (WTAR)

The WTAR (The Psychological Corporation, 2001) is a vocabulary measure, developed to estimate overall intellectual functioning. The number of correct pronunciations is calculated and using the provided norms, a scaled score and predicted full scale intelligence quotient (PFSIQ) are generated. The PSFIQ was utilized in this study as a way of determining whether the two groups were similar in intellectual functioning.

#### Auditory tasks

For all the auditory tasks used in this study, Presentation® software (Neurobehavioral Systems) was utilized via laptop computer, using headphones. Three tone discrimination tasks (TDTs) were created to assess individuals' ability to perceive differences in pitch (TDT-P), amplitude (TDT-A), and duration (TDT-D). They were closely modeled on TDTs from earlier studies (Strous et al., 1995; Leitman et al., 2005). There were 144 pairs of tones presented in both the TDT-A and TDT-D tasks, and 143 pairs of tones presented in the TDT-P.

For each of the TDTs, the initial tone in each pair was always set at 70 dB, 150 ms in duration, and had a frequency of 1500 Hz. The second tone within each pair was presented 500 ms after the initial tone. The second tone was either identical to the first, or increased or decreased by 2, 5, 10, 25, and 50% for that acoustic element (e.g., duration). Based on this information, the range of amplitude for the differing tones in the TDT-A varied from 35 to 105 dB. For the TDT-D, duration of differing tones ranged from 75 to 225 ms in length. The pitch of differing tones in the TDT-P varied from 750 to 2250 Hz. The variables used for analysis was accuracy (measured as a percentage correct) for the same condition and each of the abovementioned levels of deviation, leaving six variables for each TDT. Participants were required to identify whether they believed each pair of tones were the same or different, using allocated keys on the laptop keyboard.

#### Affective identification task (AIT)

The AIT was developed to assess participants' ability to identify emotion based on AP. It consisted of 24 semantically neutral sentences (e.g., “The window is made of glass”) which were spoken by both male and female actors (12 sentences per gender) in one of the following emotions: happy, sad, fearful, and neutral. Each sentence is approximately 3 s long. Sentences were presented in a randomized order. During the AIT, participants were required to indicate which emotion they believed the sentence was spoken in from the above options by pressing the corresponding key on the keyboard. Participants were measured on accuracy (percentage of correct guesses) and RT (ms) for each of the four emotions.

#### Continuous performance tasks—identical pairs version (CPT-IP)

Attention was measured using CPT-IP (Marder and Fenton, 2004) which was administered via laptop. The CPT-IP was divided into 3 s, where participants were asked to look for identical pairs in numbers that were two, three, and four digits long, with 150 trials per condition (30 hits, 30 false alarms, and 90 random numbers). The “on” time for each stimulus was 50 ms, with a dark time between stimuli of 950 ms. During the CPT-IP, participants were required to respond by clicking the mouse whenever they saw two identical numbers flash on the screen consecutively. An age and gender corrected *T*-score was generated for this task and was the variable used in the analyses.

### Procedure

Once screening was complete (MINI), participants completed a demographics questionnaire measuring participants' gender, age, date of birth, whether they had a relative with a psychiatric disorder, educational and employment information. Auditory tasks were alternated with the other questionnaires and the CPT to avoid participant fatigue. All of the activities undertaken to complete this paper was approved by the La Trobe University Faculty of Science, Technology and Engineering Human Ethics Committee, Approval Number FHEC09/R71, and the University of Melbourne Human Research Ethics Committee, Approval Number 0714996.1.

### Data analysis

#### Data screening and normality

Each of the variables was screened for normality using *z*-scores of the skewness and kurtosis levels. A single outlier, in years of education, was reduced to the maximum score of three interquartile ranges from the mean. LSHS-HF violated assumptions of normality and was transformed using log transformation. Untransformed means are presented in tables for ease of interpretation.

For the TDTs, scores were derived for the degree of difference between tones, (collapsing data from increased and decreased deviation levels), expressed as 6% level differences for each TDT. Each of the TDTs had a violation of normality for at least one of the percentage levels. The data were converted to error scores, and log-transformation of these scores successfully normalized the data to meet the assumptions required for the analyses.

The AIT overall accuracy variables for happy, sad and fear all violated normality and were log-transformed. No transformation normalized neutral sentences, and untransformed variables were used.

#### Analysis

Demographic variables were compared across groups using analysis of variance (ANOVA) or Chi squared, as appropriate. For each TDT, there were a total of 6% levels of difference (same, 2, 5, 10, 25, and 50%), with 2 and 5% representing the most difficult conditions. To explore group-based differences across the whole task, and to examine for interactions with different levels, each of the TDTs were analyzed using a 2 × 6 mixed design ANCOVA with group as the between subjects factor and degree of difference between tones as the within subjects factor. Given that age was shown to be significantly different across the groups, age was included as a covariate. Further, significant differences reported on the ANCOVAs were followed up with *post-hoc* One-Way analyses of variance. These ANCOVAs were conducted at each percentage level to determine where differences were occurring, with age being again used as a covariate. Similarly, to examine for group differences across the different emotional categories on the AIT, for the accuracy data, a 2 × 3 mixed design ANCOVA was conducted for happy, sad, and fear, with group being a between subjects factor and emotion being a within subjects factor, with age again being used as a covariate. An independent samples *t*-test was used to explore group differences for the neutral condition of the AIT for accuracy (due to the violated normality as noted above). For RTs, a 2 × 4 mixed design ANCOVA was used. Bonferroni corrections were not adopted to account for multiple comparisons for the TDT and AIT data, due to the small sample sizes. Mean effect sizes and observed power were calculated to aid interpretation of results. As noted in the introduction, group differences when examining RT data, could be due to poor attention. Therefore, a One Way ANCOVA was conducted to examine for group differences on the CPT task, which was followed up with a correlation between CPT RT and the overall RT on the AIT.

To explore the relationship between acoustic processing and AP, a correlation was conducted on the entire sample using deviation levels for the three TDTs (same, 2, 5, 10, 25, and 50%) and accuracy on the four emotions of the AIT. To further confirm whether relationships between variables were related to AVH or psychosis proneness, LSHS-HF and O-LIFE-NH were included. These correlations are reported in Table 4; they are presented to allow description of possible relationships and it is acknowledged that they have not been corrected for multiple comparisons.

The variable that was most highly correlated with LSHS-HF in the abovementioned correlational analysis was then entered into a hierarchical regression for the whole sample. Age and group were entered at the first step, and appropriate TDT variable was entered at the second step to assess whether acoustic processing predicted AVH proneness. An identical hierarchical regression was conducted using O-LIFE-NH instead of LSHS-HF to further establish whether acoustic processing deficits also predict psychosis proneness, or are specific to AVH proneness.

## Results

### Demographics

The proportion of males and females did not differ between the groups, χ^2^ (1, *N* = 52) = 2.43, *p* = 0.12, but age was found to differ significantly between the groups, *t*_(50)_ = −2.20, *p* = 0.033, so age was entered as a covariate for subsequent analyses. Demographic variables are presented in Table 1 below; they reveal that the groups were matched according to the number of years in education and PSFIQ.

**Table 1:** **Mean (standard deviation) of demographic characteristics for controls and relatives**.

	**Controls**		**Relatives**			
	***n***	***M*(*SD*)**	***n***	***M*(*SD*)**	***F***	***p***
Age	33	36.79 (13.72)	19	46.05 (16.12)	−4.84	0.033
EdYears	32	17.06 (3.18)	19	17.05 (3.08)	0.00	0.953
PSFIQ	33	109.21 (5.69)	19	107.11 (6.75)	2.93	0.093
LSHS-HF (max 12)	33	0.52 (1.18)	18	1.00 (1.64)^#^	3.59	0.064
UnEx	32	3.5 (4.34)	18	3.33 (2.43)^#^	1.53	0.222
CogDis	32	6.66 (6.1)	18	8.78 (6.92)	2.82	0.100
IntAn	32	3.94 (2.46)	18	7.17 (5.11)^†^	−6.35	0.020
ImpNon	32	6.19 (3.44)	18	6.11 (4.07)	0.04	0.851
STA	32	7.97 (6.51)	18	9.11 (5.65)^#^	2.34	0.133

The LSHS score is the hallucination sub-factor discussed previously.

# One-Way ANCOVAs for these variables were conducted using log transformed scores due to violation of normality. Non-transformed scores are presented for ease of interpretation.

† Analysis was performed using independent samples t-test. The statistic was squared to aid interpretation.

UnEx, Unusual Experiences; CogDis, Cognitive Disorganisation; IntAn, Introvertive Anhedonia; ImpNon, Impulsive Non-conformity; STA, Schizotypy.

### Auditory processing

Table 2 displays the mean error rates across the three TDT tasks.

**Table 2:** **Error rates for controls and relatives across each percentage level difference in pitch, amplitude, and duration on the tone discrimination task**.

**Percentage level (%)**	**Controls**		**Relatives**					
	***M***	***SD***	***M***	***SD***	***F***	***p***	***D***	***OP***
**PITCH**								
Same	4.75	4.12	6.53	8.56	2.54	0.118	−0.26	0.26
2	15.36	17.63	19.96	13.51	3.21	0.080	−0.29	0.27
5	9.58	15.85	10.15	12.91	0.09	0.768	−0.04	0.07
10	7.94	14.25	8.77	13.17	0.00	0.972	−0.06	0.08
25	1.04	2.59	0.44	1.31	1.30	0.261	0.29	0.27
50	0.14	0.80	0.00	0.00	1.02	0.318	0.25	0.22
**AMPLITUDE**								
Same	8.79	10.41	6.88	7.20	1.21	0.276	0.21	0.22
2	50.30	18.35	62.30	17.84	4.56	**0.038**	−0.66	0.75
5	38.33	17.80	49.78	16.13	5.00	**0.030**	−0.67	0.77
10	28.33	18.65	41.89	20.95	5.49	**0.024**	−0.68	0.78
25	12.04	12.93	24.06	26.21	3.96	0.053	−0.58	0.66
50	2.53	3.98	5.7	12.05	0.12	0.733	−0.35	0.34
**DURATION**								
Same	8.18	7.65	3.51	6.10	2.79	0.102	0.67	0.77
2	47.10	19.14	56.58	17.25	1.86	0.180	−0.52	0.58
5	50.22	15.05	60.53	12.53	3.92	0.054	−0.74	0.84
10	56.32	18.72	66.88	19.62	1.21	0.278	−0.55	0.62
25	17.26	16.89	27.85	20.51	4.33	**0.043**	−0.56	0.63
50	5.51	10.46	5.70	10.96	0.29	0.595	−0.02	0.06

The analyses for this data were performed on log-transformed error-rates and controlled for age. The figures presented here are the untransformed error-rates to aid in interpretation. Bold numbers indicate that relatives made significantly more errors in identification of different tones than controls.

OP, observed power.

#### Pitch discrimination

There was a main effect for degree of difference between tones *F*_(5, 44)_ = 17.42, *p* < 0.001, but no main effects were observed for age [*F*_(1, 48)_ = 1.72, *p* = 0.196] or group [*F*_(1, 48)_ = 0.53, *p* = 0.470]. There was an interaction between degree of difference between tones and group, *F*_(5, 44)_ = 3.05, *p* = 0.019, with relatives making more errors than controls for the more difficult deviation levels and fewer errors than controls for the easier deviation levels. An interaction between degree of difference between tones and age *F*_(5, 44)_ = 2.54, *p* = 0.042, was also observed. *Post-hoc* analyses established a trend difference at 2%, where relatives appear to make more errors than controls (see Table 2).

#### Amplitude discrimination

Results from the mixed design ANCOVA reveal that there was a within subjects main effect for the degree of difference in tone amplitude, *F*_(5, 40)_ = 16.91, *p* < 0.001, but no overall main effect for group or age were observed, nor were any interaction effects observed.

However, *post-hoc* follow-up One Way analyses of variance indicated that controls and relatives differed when discriminating between tones that differed by 2, 5, and 10%, with group differences approaching significance at 25%.

#### Duration discrimination

The mixed design ANCOVA for the TDT-D revealed a within subjects main effect for degree of difference in duration *F*_(5, 40)_ = 8.52, *p* < 0.001, but no between subjects main effects for group or age. Trends for interactions between degree of difference between tones and both group [*F*_(5, 40)_ = 2.27, *p* = 0.065] and age [*F*_(5, 40)_ = 2.16, *p* = 0.078] were observed. Table 2 illustrates relatives made more errors from 2 to 25%, although this was only significant at 25%, with a trend toward significance at 5%.

### Affective prosody

#### Accuracy of emotion identification

**Figure 1:**
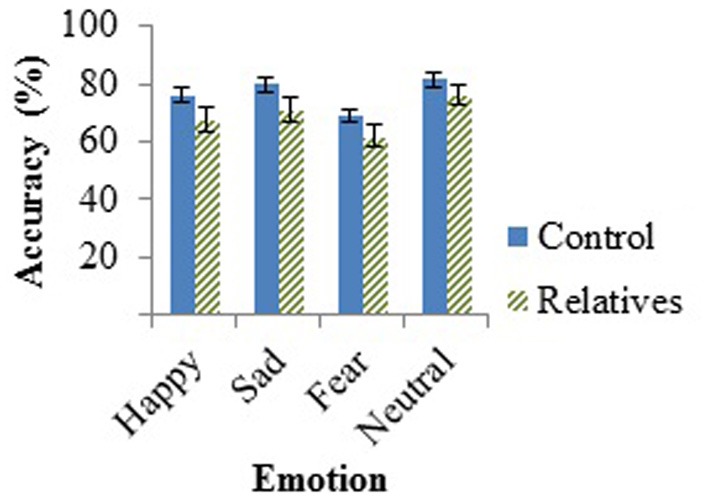
**Mean accuracy of controls and relatives for each emotion tested in the AIT, with standard error of the mean (SEM) bars**.

The hypothesized effect of group fell outside statistical significance, *F*_(1, 46)_ = 2.71, *p* = 0.106, as did the effect for emotion, *F*_(2, 45)_ = 2.85, *p* = 0.068. No interactions were found between emotion and age [*F*_(2, 45)_ = 1.52, *p* = 0.230] or emotion and group [*F*_(2, 45)_ = 0.50, *p* = 0.612]. The *t*-test performed on the neutral emotion on the AIT revealed no group differences in the ability to detect neutral sentences, *t*_(47)_ = −1.36, *p* = 0.18. Group performances on the AIT, measured by accuracy, can be found in Figure 1 below.

#### Reaction time for emotion identification

For emotion identification RT, there was a significant main effect of group, *F*_(1, 46)_ = 10.77, *p* = 0.002, as well as a main effect of age, *F*_(1, 46)_ = 11.39, *p* = 0.002. There was no main effect for emotion, *F*_(3, 44)_ = 1.31, *p* = 0.282, although there were trends for an interaction between emotion and group, *F*_(3, 44) = 2.25_, *p* = 0.095, and an interaction between emotion and age, *F*_(3, 44)_ = 2.38, *p* = 0.082. Group mean RTs for each emotion on the AIT are in Table 3. In a secondary analysis, a series of follow up One-Way ANCOVAs were conducted on RT for each of the four emotions. The relatives performed slower than controls in the happy [*F*_(1, 49)_ = 6.53, *p* = 0.014] and neutral [*F*_(1, 49)_ = 11.80, *p* = 0.001] conditions.

**Table 3:** **Mean, standard deviation and effect sizes of RT (ms) for controls and relatives for each of the four emotions of the AIT**.

	**Controls**		**Relatives**					
	***M***	***SD***	***M***	***SD***	***F***	***p***	***d***	***OP***
Happy	874.25	254.84	1143.90	331.37	6.53	**0.014**	−0.91	0.80
Sad	831.78	172.40	970.87	261.94	2.39	0.129	−0.63	0.87
Fear	1051.27	283.18	1212.74	299.10	1.14	0.291	−0.55	0.93
Neutral	783.05	205.97	1038.41	280.36	11.80	**0.001**	−1.04	0.90

Age controlled for as a covariate in each analysis. Bold numbers indicate that relatives made significantly more errors in identification of different tones than controls.

#### Attention

A univariate ANCOVA revealed that group differences in attention approached significance, *F*_(1, 31)_ = 3.99, *p* = 0.056, with relatives exhibiting lower *T*-scores (*M* = 42.24) than controls (*M* = 48.86). A correlation analysis was conducted between attention and RT on the AIT to determine whether slower RT was associated with impaired attention on the CPT; no correlations were significant.

#### Relationship between the variables

A further correlation analysis was conducted to examine the relationships between acoustic processing and AP, and the relationships between acoustic processing and AVH and psychosis proneness (Table 4). The correlation was conducted on the entire sample.

**Table 4:** **Descriptive correlations between error rates for variables in the TDT-A, TDT-D, TDT-P, the four emotions of the AIT, hallucination proneness, and psychosis proneness**.

	**Amplitude**						**Duration**						**Pitch**					
	**0%**	**2%**	**5%**	**10%**	**25%**	**50%**	**0%**	**2%**	**5%**	**10%**	**25%**	**50%**	**0%**	**2%**	**5%**	**10%**	**25%**	**50%**
Happy	−0.11	0.35^*^	0.37^*^	0.49^***^	0.38^**^	0.20	−0.03	0.14	0.06	0.09	0.23	0.08	−0.00	0.33^*^	0.28	0.24	0.12	0.08
Sad	0.10	0.00	−0.01	0.02	0.10	0.22	0.23	−0.02	0.04	0.03	0.04	−0.06	0.15	0.05	0.02	0.06	−0.08	−0.07
Fear	0.19	0.05	−0.10	0.00	0.05	0.20	−0.11	−0.08	−0.14	0.03	0.27	0.16	0.20	0.07	0.24	0.15	0.07	0.17
Neutral	−0.05	0.11	0.37^*^	0.49^***^	0.38^**^	0.20	−0.03	0.14	0.01	0.25	0.22	0.16	0.23	0.20	−0.02	0.05	0.05	−0.01
LSHS-HF	0.22	0.15	0.17	0.20	0.21	0.06	−0.03	0.32^*^	0.26	0.19	0.28	0.17	−0.06	0.44^***^	0.29^*^	0.32^*^	0.34^*^	−0.06
O-LIFE-NH	0.03	−0.07	0.17	0.20	0.21	0.06	−0.03	0.32^*^	0.10	0.18	0.20	0.08	−0.11	0.25	0.22	0.10	0.08	−0.11

The correlation analysis was conducted on error rates for the four emotions and for each variable from the three TDTs.

* p < 0.05;

** p < 0.01;

*** p < 0.001.

This analysis indicates that recognition accuracy for happy and neutral sentences was moderately correlated with accuracy in amplitude discrimination (2, 5, 10, and 25%). Recognition accuracy for happy sentences was also correlated with pitch discrimination at the 2% level. Affective identification appeared unrelated to duration discrimination. Variables measuring AVH and psychosis proneness are also presented in Table 4. Pitch discrimination was specifically predictive of AVH proneness (LSHS-HF), whilst the 2% condition of the TDT-D was correlated with both AVH and psychosis proneness (O-LIFE-NH).

As AVH proneness was most significantly correlated with the TDT-P at 2%, this variable was chosen as the best predictor to enter into the regression analyses. The regression analysis conducted for AVH proneness is found in Table 5 below. This analysis reveals that both age and group, and TDT-P 2% significantly predict AVH proneness, with the model found to be significant, *F*_(3, 46)_ = 5.74, *p* = 0.002 and accounting for 23% of the total variance. A similar regression was performed using the same predictor variables and the O-LIFE-NH. This model was not significant.

**Table 5:** **Hierarchical multiple regression analysis predicting hallucination proneness from acoustic processing**.

		**Hallucination proneness**			
**Predictor**	**B**	**Adjusted *R*^2^**	**Δ*R*^2^**	**Δ*F***	**Δ*p***
Step 1		0.10	0.13	3.57	0.036^*^
Age	−0.33				
Group	0.28				
Step 2		0.23	0.14	8.87	0.005^**^
Age	−0.43				
Group	0.18				
TDT-P 2%	0.41				

The dependent variable measuring hallucination proneness was the LSHS hallucination factor extracted in accordance with Laroi and Van der Linden (2005).

* p < 0.05;

** p < 0.01.

## Discussion

The aim of the current study was to empirically investigate acoustic processing and AP in a sample of controls and relatives. Further, this study aimed to determine whether pitch perception was related to AP, and whether pitch perception predicted AVH proneness. We present preliminary data addressing these aims.

In examining acoustic processing, our main hypothesis was that relatives would make significantly more errors in pitch discrimination on the TDT-P. This hypothesis was not supported. The literature regarding amplitude and duration discrimination is more limited, therefore, no predictions were made with regards to differences between controls and relatives on both the TDT-D and TDT-A. However, groups were shown to differ on the TDT-A at 2, 5, and 10%, whilst they differed on the TDT-D at 25%.

Pitch perception is the element of acoustic processing most consistently found to be impaired in schizophrenia patients (Rabinowicz et al., 2000; Leitman et al., 2005, 2007, 2010b; Matsumoto et al., 2006; Phillips, 2009; Kantrowitz et al., 2013), so it was unexpected that it was on this task that there was least evidence for differences in relatives. However, there may be differences in difficulty between the tasks of pitch, duration and amplitude discrimination. Indeed, first episode and chronic schizophrenia patients only tend to show difficulties in pitch discrimination below 3–5% differences in pitch (Rabinowicz et al., 2000). Given that the extent of deficits may be smaller still in relatives, it may have been that the pitch discrimination task used was insufficiently sensitive to pick up difficulties.

The current TDT-P was closely based on that by Leitman et al. (2005) and Strous et al. (1995). Leitman et al. used three sets of base frequencies of 500, 1000, and 2000 Hz to help avoid learning effects; an approach that has since been replicated (Rabinowicz et al., 2000; Leitman et al., 2006, 2010a; Kantrowitz et al., 2013). The current study utilized a base frequency of 1500 Hz, which is halfway between Leitman's middle and upper base tones. Further, other studies utilized a base frequency of 1000 Hz for their standard tone (Strous et al., 1995; Javitt et al., 2000). It may be that subtle differences are much easier for participants to detect at a higher frequency of 1500 Hz, rather than 500 or 1000 Hz.

Nonetheless, we found a statistically significant effect for duration. Group differences were found between controls and relatives when required to discriminate between tones altered in duration where the tones differed by 25%. Effect sizes were either moderate or moderate-to-large for every condition except for 50%. Duration is one of the key acoustic processes involved in AP (Leitman et al., 2010a) and, based on these findings, duration may be important to explore in schizophrenia patients and relatives. The finding of group differences for tone discrimination based on duration is consistent with the literature showing that schizophrenia patients have reduced amplitude MMN in response to duration deviant stimuli (Baldeweg et al., 2002; Umbricht and Krljes). There is also some evidence to suggest that reduced amplitude duration MMN can also be found in those at high risk for schizophrenia (Shin et al., 2009) and relatives of schizophrenia patients (Michie et al., 2002; Sevik et al., 2011). More research needs to be conducted to determine the role of impaired duration discrimination in predisposition to AVHs.

The current results also showed that relatives differed from controls in their ability to detect subtle differences in amplitude between two tones at 2, 5, and 10%. To our knowledge, no one has investigated amplitude discrimination in schizophrenia patients before, let alone relatives with either behavioral or neurophysiological methods. The findings from this study suggest that this area warrants further investigation to ascertain the role of amplitude perception in schizophrenia patients and possibly specifically in AVHs.

Pitch perception, as previously described, is involved in the contextual encoding of auditory information when perceiving or re-experiencing verbalizations, and is particularly relevant to decoding the affective meaning of speech. Duration and amplitude also play a role in these mechanisms. Leitman et al. (2010b) have previously outlined how combinations of these aspects of sound contribute to each emotion. The effect sizes observed in this study suggest each of these processes warrant further investigation in both schizophrenia patients and their relatives. Perhaps deficits in all three areas are underlying patients' difficulty in perceiving information accurately during encoding, as well as assigning the appropriate source to inner thought or re-experienced events.

Relatives were expected to have greater difficulty in emotion perception on the AIT, which was expected to be reflected in lower accuracy scores. Although relatives appeared to have higher error rates than controls on the AIT, there were no significant effects for group or emotion. This is inconsistent with schizophrenia research which has found patients to perform worse than controls on happy, fear, and neutral sentences (Leitman et al., 2010b). Relatives' slower RT on the AIT for happy and neutral sentences suggests that RT may be a more sensitive variable. Further research with a greater number of participants needs to be conducted to ascertain whether AP perception deficits are confined to schizophrenia patients or whether they are also present in relatives.

One criticism of AP tasks is that simulated portrayals of emotion are stereotypic with exaggerated differences between various emotions (Edwards et al., 2002). In everyday situations, natural emotions are conveyed by context, the content of utterances, and the speaker (Edwards et al.). Therefore, difficulties in recognizing specific emotions may not be identified in AP tasks due to the exaggerated nature of the emotions presented, making them easier to detect. Naturally recorded emotions may be a more effective way at assessing individuals' abilities to recognize and distinguish between various emotions. Furthermore, in our study, participants appeared to be performing at close to ceiling on the AIT, with both groups displaying mean error rates of between 2 and 4%. This suggests that perhaps the artificial nature of the task made it easy for participants to identify the emotion, and thus the task was not sensitive enough to distinguish the two groups.

Whilst relatives did not differ significantly from controls in their ability to accurately identify emotions, they did take significantly longer to do so. Results from the AIT indicated that relatives were slower at identifying happy and neutral sentences, with effect sizes supporting this finding. Decreased performance for sad sentences approached significance. This supports previous findings linking AP deficits in schizophrenia to impaired recognition of sadness (Murphy and Cutting, 1990; Edwards et al., 2001; Rossell and Boundy, 2005; Bozikas et al., 2006; Leitman et al., 2010a), happiness and neutral sentences (Leitman et al., 2010a), thus suggesting that relatives display some impairment in their ability to perceive AP cues. Interestingly, happiness is associated with high levels and variability of pitch and amplitude, whilst sadness is associated with low levels and variability in amplitude and low levels of pitch (Leitman et al., 2010a), and neutral sentences can often be the most difficult to detect as they use medium levels of pitch and amplitude variability and can be very person specific. Thus, happy sentences should have been the easiest to identify, even in the presence of amplitude and pitch difficulties in the relatives. Therefore, it is unclear, based on the current data, why such “emotion” specific reaction time differences are present. Further work is needed confirming these deficits.

Impaired attention has previously been theoretically linked with impaired RT (Nuechterlein, 1977) and thus it was important to ascertain whether relatives' RTs on the AIT were related to impaired attention (lower *T*-scores), or whether RT reflected task difficulty for relatives. Results showed that attention was not significantly correlated with RT for any of the emotions, indicating no systematic relationship between these factors. This is consistent with Kee et al. (1998) who found no relationship between attention/vigilance measured by the CPT and AP. The direction of our findings and Kee et al.'s findings, suggest that RTs on AP measures for patients and relatives are less related to impaired attention, and likely mediated by their difficulty in emotion discrimination. Additionally, future research may benefit from investigating auditory attention, specifically as the CPT is a visual attention task. It maybe the lack of relationship between attention scores and the RT on the AP task were due to modality differences.

TDT-P at 2% and a number of levels of the TDT-A were found to be positively correlated with happy sentences, and the 5, 10, and 25% conditions of the TDT-A were also correlated with neutral sentences. Interestingly, happy and neutral sentences were the two conditions of the AIT where relatives displayed slower reaction times. Therefore, higher error rates on the TDT-A and TDT-P appear to be associated with increased error rates for happy and neutral sentences of the AIT. This suggests that acoustic processing deficits underlie deficits in AP perception, and supports the link between pitch perception and AP previously highlighted by Leitman et al. (2005).

Hallucination proneness was predicted to be positively correlated with error rates on the TDT-A, TDT-D and the TDT-P. Results showed that the TDT-D at 2% was weakly positively correlated with AVH proneness in the entire sample. Further, the TDT-P was positively correlated with AVH proneness at 4% levels, with the strongest positive correlation (moderate in strength) at 2%. Therefore, pitch perception appears to be closely related to AVH proneness.

In the current study, pitch perception was shown to be linked with AP perception, with AP perception previously found to be more impaired in AVH schizophrenia patients than non-AVH patients (Rossell and Boundy, 2005; Shea et al., 2007). Further analysis revealed that performance on pitch discrimination when tones differ by 2% appears to predict AVH proneness in the current sample. Further, pitch discrimination did not significantly predict psychosis proneness when hallucinatory factors have been removed from the proneness measure. This provides support for basic acoustic processing deficits, particularly with pitch perception, predicting higher order processes such as AP perception, leading to the experience of AVHs. This supports previous findings where pitch alterations of auditory stimuli increased the likelihood of schizophrenia patients attributing their recorded voice to an external source (Johns and McGuire, 1999; Johns et al., 2001, 2006). Patients appear to have difficulty extracting sufficient auditory cues of speech, which may contribute to their difficulty recognizing their own voice, or misattributing the sources of auditory verbal stimuli.

The most obvious limitations of the current investigation are those of a small sample size, and unequal group sizes. Thus, our data can only be classified as preliminary. For example, the small sample size prevented us from running separate regressions for controls and relatives when exploring the link between pitch perception and hallucination proneness. It is likely that if this analysis was re-run separately for each group, with an increased sample size, the model predicting hallucination proneness would likely have explained more of the variance for relatives than for controls. Furthermore, no corrections were made for the number of secondary analyses conducted for the TDTs or AIT, increasing the likelihood of a Type II error. Nonetheless, a number of promising results were recorded and effect sizes were calculated to support effects that were detected. Furthermore, the description of endophenotypes for schizophrenia outlined by the Consortium on the Genetics of Schizophrenia (Gur et al., 2007) suggests that endophenotypes for schizophrenia yield small to moderate effect sizes between relatives and controls, which were indeed observed here. Thus, it is highly likely that increased sample sizes in future studies would provide further evidence for the auditory deficits observed here, and perhaps would increase the likelihood of observing group differences for other neurocognitive domains such as processing speed. In addition, it is recommended that future work complete a detailed clinical interview with the patients of the relatives being studied. This will allow for a detailed history of the exact phenomenology of AVH experienced by the patients, including frequency and types of voices experienced.

The current investigation decided to utilize parents, siblings and offspring of schizophrenia patients with AVHs in the first-degree relatives group, which is consistent with each of these groups being used in the previous literature [siblings (Condray and Steinhauer, 1992; Leppanen et al., 2008; Erol et al., 2010), offspring (Erlenmeyer-Kimling and Cornblatt, 1978; Nuechterlein, 1983), and parents (Appels et al., 2003; Anselmetti et al., 2009)]. Erol et al. (2010) have critiqued the use of mixed relatives samples used in previous research (Toomey et al., 1999) but stopped short of explaining why. The use of siblings and offspring appears acceptable given strong research of genetic predisposition to schizophrenia (Matthysse and Kidd, 1976; Cannon et al., 1998; Allen et al., 2008). However, the use of parents in samples of relatives could be considered risky. When recruiting parents, one cannot be certain that the parent with the genetic predisposition to schizophrenia has been chosen. Therefore, it can be proposed that the results of the current investigation may have been strengthened if the relatives sample comprised only siblings and offspring.

We suggest possible endophenotypes in relatives of schizophrenia patients with AVHs compared with controls. However, these skills need to be investigated in first degree relatives of schizophrenia patients who have never experienced AVHs. If no evidence of these deficits is found in the second schizophrenia relative cohort, then it is reasonable to suggest that the endophenotypes that this investigation has potentially uncovered are indeed related to AVHs specifically, and are not endophenotypes for schizophrenia in general.

## Conclusion

The results from the current investigation contribute to the literature regarding endophenotypes in schizophrenia. Impaired pitch perception was related to slower performance on the AIT and predicted AVH proneness in relatives, suggesting it may be a potential endophenotype for AVHs in schizophrenia and strengthens the argument that auditory processing is a fruitful area to investigate in endophenotype research. The endophenotypes identified are, as far as we are aware, the first to be investigated in relation to AVHs specifically. More research needs to be conducted to determine whether these endophenotypes are limited to relatives of patients with AVHs, or whether they are present in all schizophrenia relatives. To further confirm that pitch perception deficits are related to predisposition to AVHs specifically, it would be prudent to conduct a similar study that includes AVH and non-AVH patients, relatives of AVH and non-AVH patients, and controls. Confirmation of an endophenotype for AVHs centered on acoustic processing could lead to the establishment of assessment and pre-screening of individuals, to identify those who are at increased risk of developing AVHs, thus adding to the prevention and early intervention approach already shown to be successful in the treatment of schizophrenia.

### Conflict of interest statement

The authors declare that the research was conducted in the absence of any commercial or financial relationships that could be construed as a potential conflict of interest.
